# The prevalence and isolated subtypes of BK polyomavirus reactivation among patients infected with human immunodeficiency virus-1 in southeastern China

**DOI:** 10.1007/s00705-018-3724-y

**Published:** 2018-02-12

**Authors:** Caiqin Hu, Ying Huang, Juwei Su, Mengyan Wang, Qihui Zhou, Biao Zhu

**Affiliations:** The Department of Infectious Diseases, State Key Laboratory for the Diagnosis and Treatment of Infectious Diseases, Collaborative Innovation Center for the Diagnosis and Treatment of Infectious Diseases, the First Affiliated Hospital, College of Medicine, Zhejiang University, Hangzhou, China

## Abstract

BK polyomavirus (BKPyV) is an opportunistic infectious pathogen that is associated with hemorrhagic cystitis and nephropathy, mainly in transplant recipients and human immunodeficiency virus 1 (HIV-1) infected patients. However, molecular characterization studies of BKPyV in China are rare. This study was designed to elucidate the prevalence and to determine the main subtypes of BKPyV among HIV-1-infected patients in southeastern China. In addition, the increased incidences for BKPyV reactivation were analyzed. The isolated BKPyV DNA was amplified by polymerase chain reaction (PCR) and the specimen sequences were aligned with the reference sequences for phylogenetic analysis. In this study, BKPyV viruria was detected in 64.2% (88/137) of HIV-1-infected patients. Patients in the BKPyV-positive group were more diverse with respect to gender (*P* = 0.039) and age (*P* = 0.023) than their counterparts in the BKPyV-negative group, and they had a higher rate of co-infection with tuberculosis (TB) (*P* = 0.026). Viruria was more commonly found in patients with CD4 counts <200 cells/mm (72.7%) than in those with CD4 counts ≥200 cells/mm (58.5%) (not significant). All sequenced BKPyV isolates belonged to subtype I (13/32) and IV (19/32). A high prevalence of BKPyV reactivation was discovered in patients with HIV-1 infection. Females and elderly individuals, as well as those with a TB co-infection, appeared more susceptible to BKPyV reactivation in this study. BKPyV viruria was found more often and was associated with lower CD4 counts.

## Introduction

The BK polyomavirus (BKPyV), a ubiquitous pathogen, is a member of the polyomaviridae family, originally isolated from the urine of a renal allograft recipient by Gardner SD in 1971 [1]. Approximately 90% of the human population is asymptomatically infected with the BKPyV during childhood. Afterwards, the primary infection virus remains latent [2–6]. In immunocompromised patients, BKPyV reactivation often causes ureteric stenosis and severe tubulointerstitial nephritis in post-transplant kidney patients and hemorrhagic cystitis in bone marrow transplant patients [7–12]. Human immunodeficiency virus 1 (HIV-1) patients are subject to other complications, such as meningitis, encephalitis, lymphoma, retinitis, syphilis, tuberculosis (TB), and pneumonia [7, 13–15].

Different BKPyV subtypes (I, II, III and IV) have been successfully detected in some countries, including Japan, Africa, the UK, Italy, the US, and other countries [2, 3, 7, 8, 14, 16, 17]. The subtype I is further classified into subgroups I/a, I/b 1, I/b 2, and I/c, and subtype IV is further classified into subgroups IV/a 1, IV/a 2, IV/b 1, IV/b 2, IV/c 1, and IV/c 2. Subtypes II and III are rarely found in the urine [3, 8, 18–23]. Each of these subgroups harbors a unique geographic distribution: subtype I is widespread all over the world, while subtype IV is prevalent in East Asia [21, 24, 25].

There have been several studies of polyomavirus in immunocompetent adults and renal allograft recipients [3, 21, 24, 26]. However, studies concerning BKPyV-reactivation among HIV-1 patients are still limited in China. For this reason, we carried out this study to investigate BKPyV reactivation and genotypes among Chinese HIV-1 patients. In order to investigate factors influencing BKPyV reactivation, the following data from patients were taken into consideration: gender, age, CD4+ T-cell levels, highly active antiretroviral therapy (HAART) initiation, and HIV-1-associated complications.

## Materials and methods

### Study population

A total of 137 HIV-1-infected patients from the Department of Infectious Disease of the First Affiliated Hospital of Zhejiang University were admitted to participate in the study. The cohort included 115 patients who were receiving HAART and 22 patients who were not being treated with antiretroviral therapy during enrollment in the study. We clearly explained the purpose of the study to each patient and obtained informed consent from all participants.

### DNA extraction, PCR, and sequence analysis

At the beginning of the study, a midstream urine sample was collected from each patient. Ten milliliters of urine was divided into four centrifuge tubes, and each tube was centrifuged at 12,000 r/min for 10 min; the supernatant was discarded, and the remaining urinary sediment was resuspended in 100 µl sterile phosphate-buffered saline (PBS). Viral DNA was extracted with a QIAamp DNA Mini Kit (Sangon Biotech, Shanghai) in accordance with the manufacturer’s instructions.

BKPyV DNA Primers were designed to be specific to the BKPyV VP1 fragment. The forward and the reverse primer sequences were as follows: 5′-GAAGTTCTAGAAGTTAAAACTGGG-3′ and 5′-CCTATTCAAGGCAGTAATTTCCAC-3′, respectively [16, 19].

The target sequence was amplified in a reaction volume of 25 µL containing 1 µL of template, 2.5 µL of 10×PCR Buffer, each primer at a concentration of 10 µM, 0.5 µL of dNTPs, 2.5 µL of MgCl_2_, and 2.5 U of Taq polymerase per reaction.

The amplification parameters were as follows: DNA was denatured for 5 min at 95°C, followed by 35 cycles of 95°C (30 s), 55°C (35 s), and 72°C (1 min) and a final elongation step at 72°C for 8 min. DNA sequencing was carried out using an automated sequencer (ABI 3730 gene analyzer DNA sequencer). The fragment obtained was 342 bp in size.

### Phylogenetic analysis

22 BKPyV whole-genome sequences were obtained from GenBank [20, 25]. The accession numbers of the referenced sequences are shown in Table 1. Nucleotide sequence data from this study were added to the GenBank database under the accession numbers MF522142–MF522173, which corresponded to the detected nucleotide sequences ZHE-1 to ZHE-32.

**Table 1:** BKPyV reference sequences selected for phylogenetic analysis

Subtype/subgroup	Isolate	Geographic origin	GenBank accession no.
I/a	DUN	USA	NC_001538
I/a	KEN-1	Kenya	AB263926
I/b1	Dik	The Netherlands	AB211369
I/b1	WW	South Africa	AB211371
I/b2	JL	The Netherlands	AB211370
I/b2	FNL-12	Finland	AB263918
I/c	MT	Japan	AB211372
I/c	TW-1	Japan	AB211381
II	ETH-3	Ethiopia	AB263916
II	SB	UK	Z19536
III	AS	UK	M23122
III	KOM-3	Japan	AB211386
IV/a1	VNM-7	Vietnam	AB269869
IV/a1	PHL-8	Philippines	AB269859
IV/a2	MMR-1	Myanmar	AB269841
IV/b1	THK-8	Japan	AB211390
IV/b1	TW-3	Japan	AB211391
IV/b2	KOM-2	Japan	AB211387
IV/b2	JPN-15	Japan	AB269834
IV/c1	MON-1	Mongolia	AB269846
IV/c1	SWC-1	China	AB269863
IV/c2	ITA-4	Italy	AB269833

A total of 55 sequences, including 32 covering the VP1 coding region of BKPyV DNA targeted in this study, 22 BKPyV whole-genome sequences from GenBank and SA12, a baboon polyomavirus closely related to BKPyV [20, 25, 27–29]), were aligned and analyzed using BIOEDIT and MEGA software to construct a neighbor-joining (NJ) and Kimura 2-parameter model method tree. To determine the confidence level of the branching patterns in the tree, 1,000 bootstrap replicates were performed

### Statistical analysis

The independent samples non-parametric *t*-test was performed on numerical data, while chi-square test and Fisher’s exact tests were carried out for the categorical variables. *P*-values of less than 0.05 were considered as statistically significant. All data were analyzed using SPSS software, version 23.0.

## Results

### A high BKPyV reactivation rate in HIV-1-infected patients

A total of 137 HIV-infected patients (16 women and 121 men) were enrolled in the study. The mean age was 39±13 years old (Table 2). Regarding the level of immunity, the CD4+ T cell counts were divided into two groups: CD4 <200, ≥200 cells/mm^3^ and a clear divergence in median CD4 counts (48 vs. 397 cells/mm^3^) within the BKPyV-positive and BKPyV-negative groups. An obvious difference was discovered in the interior group: there was 72.7 % BKPyV viruria present in the group with CD4 counts <200 cells/mm^3^ and 58.5% where CD4 counts ≥200 cells/mm^3^.

**Table 2:** Comparison of BKPyV excretion rates among HIV-infected patients

Category	Total	No. of BKPyV negatives	No. of BKPyV positives	*P*-value
Overall	137	49 (35.8)	88 (64.2)	
Gender, n (%)				0.039^1^
Male	121	47 (38.8)	74 (61.2)	
Female	16	2 (12.5)	14 (87.5)	
Age, years, n (%)				0.023^2^
≤20	1	1 (100)	0 (0.00)	
21–40	88	34 (38.6)	54 (61.4)	
41–60	35	12 (34.3)	23 (65.7)	
>60	13	2 (15.4)	11 (84.6)	
CD4+ cells/mm, n (%)				0.089^1^
<200	55	15 (27.3)	40 (72.7)	
≥200	82	34 (41.5)	48 (58.5)	
Treatment, n (%)				
Tenofovir	61	21 (34.4)	40 (65.6)	0.769^1^
Zidovudine	43	20 (46.5)	23 (53.5)	0.076^1^
Lamivudine	111	42 (37.8)	69 (62.2)	0.296^1^
Efavirenz	88	35 (39.8)	53 (60.2)	0.190^1^
Comorbidity, n (%)				
Hepatitis B	8	3 (37.5)	5 (62.5)	NS
TB	21	3 (14.3)	18 (85.7)	0.026^1^
Lymphoma	6	2 (33.3)	4 (66.6)	NS
Cryptococcal	9	5 (55.6)	4 (44.4)	NS
meningitis				
PCP	6	3 (50.0)	3 (50.0)	NS

1. Pearson Chi-Square, Continuity Correction and Fisher’s exact test for categorical variables;

2. Independent samples *t* test for numerical data

As shown in Table 2, BKPyV viruria was detected in 88 HIV-1-infected patients (64.2%). The BKPyV-positive excretion rate was higher in women than men (87.5% vs. 61.2%, respectively; *P* = 0.039) and a direct correlation was revealed between age and BKPyV excretion (*P* = 0.023). BKPyV viruria was found in 16 of 22 (72.7%) individuals who had not undergone antiretroviral therapy compared to 72 of 115 (62.6%) of the HAART patients (*P* = 0.364). Co-infection with TB appeared linked to a higher susceptibility to BKPyV reactivation than other comorbidities, with an 85.7% BKPyV excretion rate (*P* = 0.026) (Table 2).

### Subtype IV is most prevalent in southeastern China

**Fig. 1:**
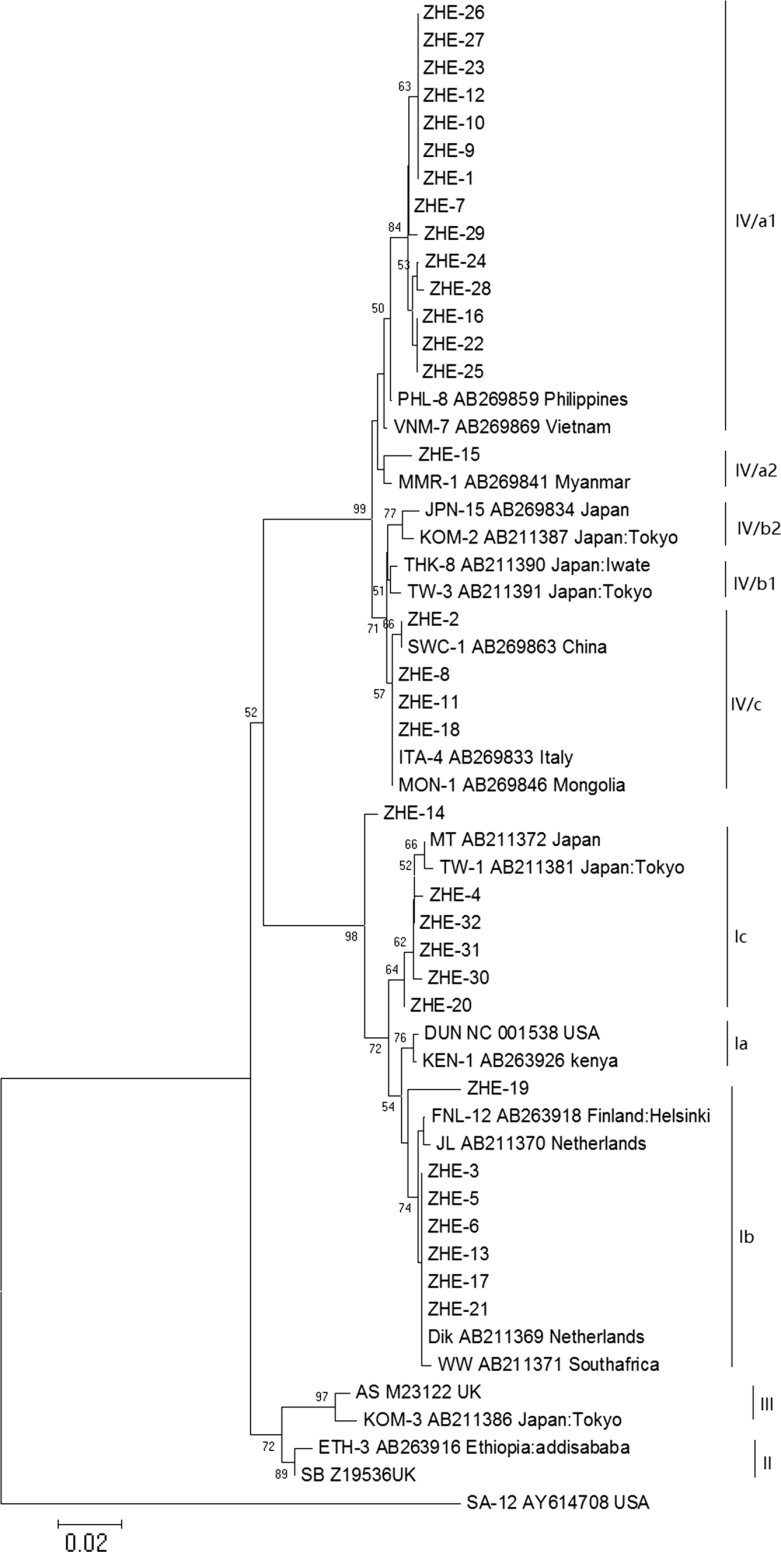
Four subtypes of BKPyV identified by phylogenetic analysis. The phylogenetic tree was constructed using MEGA version 7.0.20. Bootstrap replicates were performed 1,000 times (only the numbers at nodes above 50 are shown). The types (I, II, III, and IV) and subtypes (IV-a1, IV-a2, IV-b1, IV-b2, IV-c, Ia, Ib, and Ic) are indicated

Using genotyping and phylogenetic methods, 32 sequences were classified into four major clusters (I through IV), with the BP ranging from 89% (for subtype II) to 99% (for subtype IV) (Figure 1). Subtype I was further categorized into three sub-clusters: I/a, I/b, and I/c, with the BP ranging from 54% to 76%. Concurrently, subtype IV was divided in to five subgroups: IV/a1, IV/a2, IV/b1, IV/b2, and IV/c. Overall, the present study demonstrated that subtype IV was the most common BKPyV subtype in southeastern China (19/32), with 15 (46.88%) isolated sequences classified as IV-a and 4 (12.5%) as IV-c. Subtype I ranked second-highest in prevalence (13/32), with 6 (18.75%) isolated sequences classified as I-c and 7 (21.87%) as I-b.

## Discussion

Epidemiological data has shown that up to 90% of the human population is seropositive for BKPyV. After primary asymptomatic infection, BKPyV remains latent. However, low prevalence rates (6%–40%) in immunocompetent children and adults have been reported in previous assays [5, 13, 30, 31]. In this study, a high polyomavirus excretion rate (64.2%), detected through BKPyV DNA, was identified in the urinary tract of HIV-1 patients. This high prevalence of polyomavirus indicates that BKPyV reactivation may be linked to the T cell immunodeficiency in HIV-1-infected patients.

BKPyV viruria was more common in females and elderly HIV-1-infected patients in this study (*P* values of 0.039 and 0.023, respectively). These data are in contrast with previous studies that indicated that BKPyV excretion rates had no relationship with age or gender [5, 16, 30, 32, 33]. Meanwhile, we discovered that HIV-1 patients co-infected with TB were more susceptible to BKPyV reactivation (*P* = 0.026). TB is not an uncommon disease to find in co-infections with HIV in developing countries, and a study by Al-Warthan et al. indicated that polyomavirus nephropathy was reactivated with disseminated TB [14]. Increased BKPyV viruria was found in females, the elderly and individuals co-infected with TB. These results may only apply to this experiment due to specific demographic characteristics and the small population of patients.

BKPyV DNA was found in 58.5% of HIV-1-infected patients with CD4+counts ≥200 cells/mm^3^ and 72.7% of the patients with CD4+ counts <200 cells/mm^3^. These results also revealed that higher CD4 counts corresponded to lower BKPyV viruria. However, there were no statistically significant associations between the presence of BKPyV in urine and the degree of immunodeficiency. Other investigators have also demonstrated increased BKPyV viruria with lower CD4 counts (not significant) [2, 17]. Several reports have illustrated that BKPyV reactivation is closely associated with the elimination of virus-specific memory T cells and impairment of cell-mediated immune responses [33–37].

BKPyV isolates can be classified into four major subtypes based on molecular methods. In the present study, subtype IV was shown to be the most common BKPyV subtype in southeastern China (19/32), followed by subtype I (13/32). The majority of reports have found subtype I to be the most prevalent worldwide, while subtype IV is common in Asia and in parts of Europe [3, 21]. In China, a previous study reported that subtype I was found at the highest rate (63%) and subtype IV at the second highest rate (37%) in immunocompetent elderly patients in Shanghai [24]. Chen et al. also showed that 23 BKPyV isolates (64%) were classified as subtype I, and 13 (36%) were classified as subtype IV in Chinese renal transplant patients [3]. All these results indicate a dominance of subtype I, which differs from the present article where subtype IV was dominant.

In conclusion, this is first study to evaluate the high reactivation rates and specific subtypes of BKPyV in HIV-1 patients in southeastern China. Our findings also indicated that being female, elderly, and co-infected with TB appeared to render HIV-1 patients more susceptible to BKPyV reactivation. BKPyV viruria was also found to be more closely associated with lower CD4 counts. It remains uncertain whether higher CD4 counts is associated with elimination of BKPyV viruria, therefore further study is needed to clarify this aspect of BKPyV persistence.

## Abbreviations

BKPyV: BK polyomavirus
HIV-1: human immunodeficiency virus 1
HAART: highly active antiretroviral therapy
TB: Tuberculosis
